# Large-scale experiments into the tsunamigenic potential of different iceberg calving mechanisms

**DOI:** 10.1038/s41598-018-36634-3

**Published:** 2019-01-29

**Authors:** Valentin Heller, Fan Chen, Markus Brühl, Roman Gabl, Xuexue Chen, Guido Wolters, Helge Fuchs

**Affiliations:** 1 Environmental Fluid Mechanics and Geoprocesses Research Group, Faculty of Engineering, University of Nottingham, Nottingham, NG7 2RD UK; 2 Leichtweiß-Institute for Hydraulic Engineering and Water Resources (LWI), Department of Hydromechanics and Coastal Engineering, Technische Universität Braunschweig, Beethovenstraße 51a, 38106 Braunschweig, Germany; 3 School of Engineering, Institute for Energy Systems, University of Edinburgh, Edinburgh, EH9 3DW UK; 4 Unit of Hydraulic Engineering, University of Innsbruck, Technikerstrasse 13, 6020 Innsbruck, Austria; 5 Department of Hydraulic Engineering, Delft University of Technology, Stevinweg 1, 2628 CN Delft, The Netherlands; 6 Royal HaskoningDHV, George Hintzenweg 85, 3009 AM Rotterdam, The Netherlands; 7 Deltares, Coastal Structures and Waves, Boussinesqweg 1, 2629 HV Delft, The Netherlands; 8 Laboratory of Hydraulics, Hydrology and Glaciology (VAW), ETH Zurich, 8093 Zurich, Switzerland

## Abstract

Mass balance analysis of ice sheets is a key component to understand the effects of global warming. A significant component of ice sheet and shelf mass balance is iceberg calving, which can generate large tsunamis endangering human beings and coastal infrastructure. Such *iceberg-tsunamis* have reached amplitudes of 50 m and destroyed harbours. Calving icebergs interact with the surrounding water through different mechanisms and we investigate five; A: capsizing, B: gravity-dominated fall, C: buoyancy-dominated fall, D: gravity-dominated overturning and E: buoyancy-dominated overturning. Gravity-dominated icebergs essentially fall into the water body whereas buoyancy-dominated icebergs rise to the water surface. We find with unique large-scale laboratory experiments that iceberg-tsunami heights from gravity-dominated mechanisms (B and D) are roughly an order of magnitude larger than from A, C and E. A theoretical model for released iceberg energy supports this finding and the measured wave periods upscaled to Greenlandic outlet glaciers agree with field observations. Whilst existing empirical equations for landslide-tsunamis establish estimates of an upper envelope of the maximum iceberg-tsunami heights, they fail to capture the physics of most iceberg-tsunami mechanisms.

## Introduction

Land ice melt and retreat is one of the most visible effects of climate change and contributes ~1.5 mm/year to the global sea-level rise of a total of ~2.7 mm/year^1–3^. Mass balance analysis of ice sheets and selves is thus a key component to understand sea-level rise and the effects of global warming^1,2,4–13^. Iceberg calving accounts for most of the mass loss from the Antarctic Ice Sheet^6^ and for 32% of the Greenland Ice Sheet^8,14^ between 2009–2012 of its overall ice mass loss of approximately −269 ± 51 Gt/year^12^.

**Figure 1:**
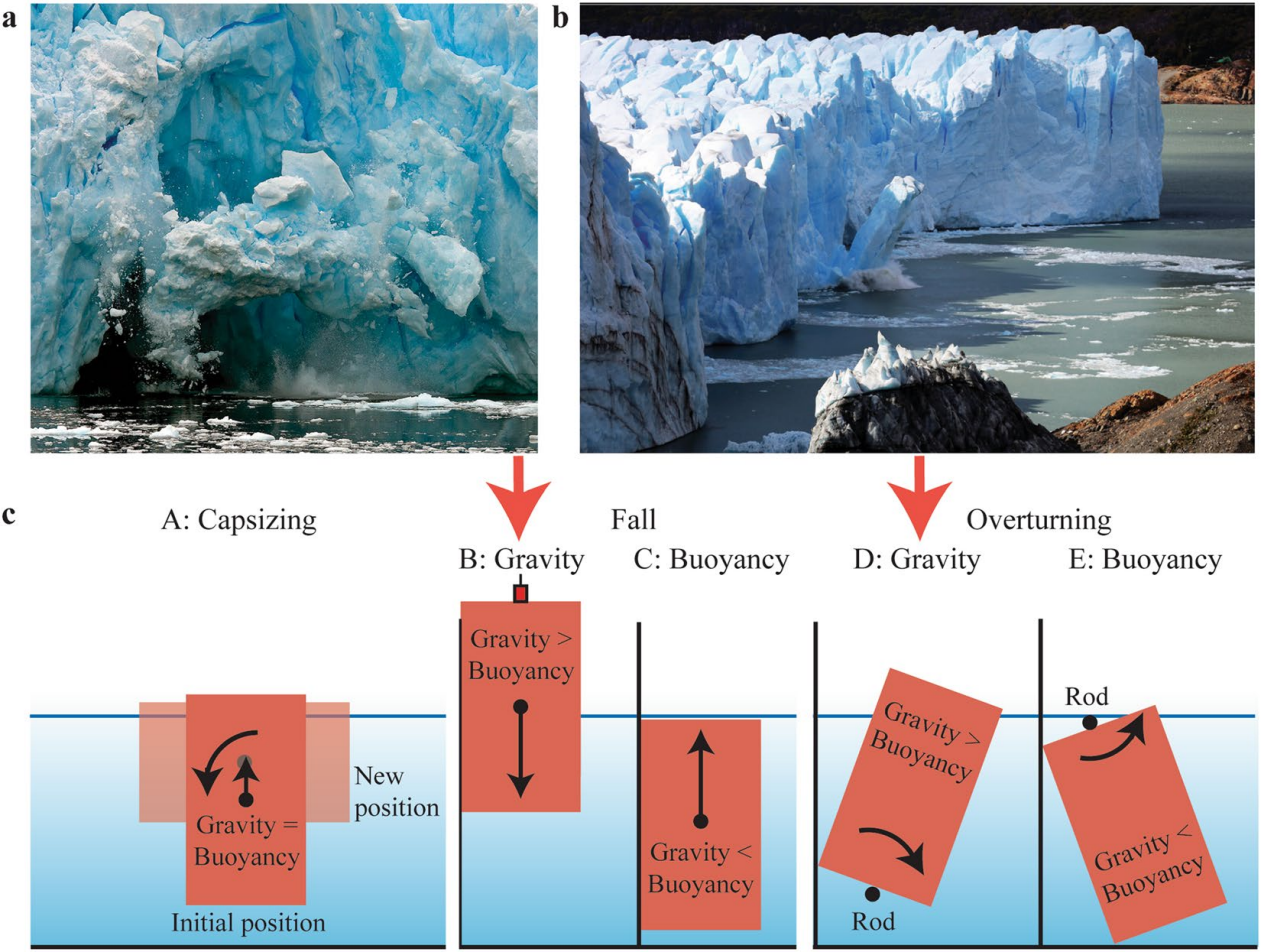
Real and idealised iceberg calving and iceberg-tsunami generation. **(a)** Falling iceberg at Neko Harbour, Antarctica (courtesy of Alek Komarnitsky - www.komar.org). **(b)** Overturning iceberg at Perito Moreno Glacier, Argentina (photograph by Victor Qixiang Chen - http://photo.qyer.com/7259134/allphoto). **(c)** Sketches of investigated idealised iceberg calving mechanisms from left to right: A: capsizing, B: gravity-dominated fall, C: buoyancy-dominated fall, D: gravity-dominated overturning and E: buoyancy-dominated overturning.

Iceberg calving is not only relevant for ice mass balance, but can also generate large tsunamis (Fig. 1). This type of waves, called *iceberg-tsunamis* hereafter (short for iceberg-generated tsunamis), is the focus of this work. Iceberg-tsunamis are typically observed in the summer season at grounded glaciers such as Helheim^5^ and Eqip Sermia^15^ and also for mountain glaciers including the Tasman Glacier^16^. Their relevance as a natural hazard and to stimulate additional iceberg calving has been highlighted in several recent studies^6,14,17–20^. Recorded iceberg-tsunamis include an amplitude of 50 m at Eqip Sermia, Greenland, destroying infrastructure in 2014^15^ and a 24 cm large wave approximately 25 km from the Helheim outlet glacier in east Greenland^21^. Of similar interest are iceberg-tsunamis generated by capsizing icebergs^18,22,23^, which may, however, generate significantly smaller waves^18^. Nevertheless, such an event destroyed a harbour in Greenland in 1995^24^. Such observed extreme events raise the question which magnitude iceberg-tsunamis may reach and how dangerous they are for human beings and our coastal infrastructure.

Figure 1a,b show iceberg calving events in nature. Depending on the initial position of the iceberg relative to the water surface and the mass kinematics, the icebergs interact with the surrounding water via different iceberg calving mechanisms^6,17,20^. We investigate the five idealised mechanisms illustrated in Fig. 1c namely A: capsizing, B: gravity-dominated fall, C: buoyancy-dominated fall, D: gravity-dominated overturning and E: buoyancy-dominated overturning. Gravity-dominated icebergs essentially fall into the water body whereas buoyancy-dominated icebergs rise to the water surface.

In this article the tsunamigenic potentials of mechanisms A to E are investigated with unique large-scale experiments conducted in a 50 m × 50 m wave basin at Deltares in Delft, The Netherlands. We quantify the maximum heights and energies of the associated iceberg-tsunamis and relate them to the theoretically released energies of the icebergs. The work further links the new results to predictive methods of landslide-tsunamis to potentially transfer knowledge from an established related research field to the relatively new field of iceberg-tsunamis.

## Methods

### Experimental set-up and conditions

**Figure 2:**
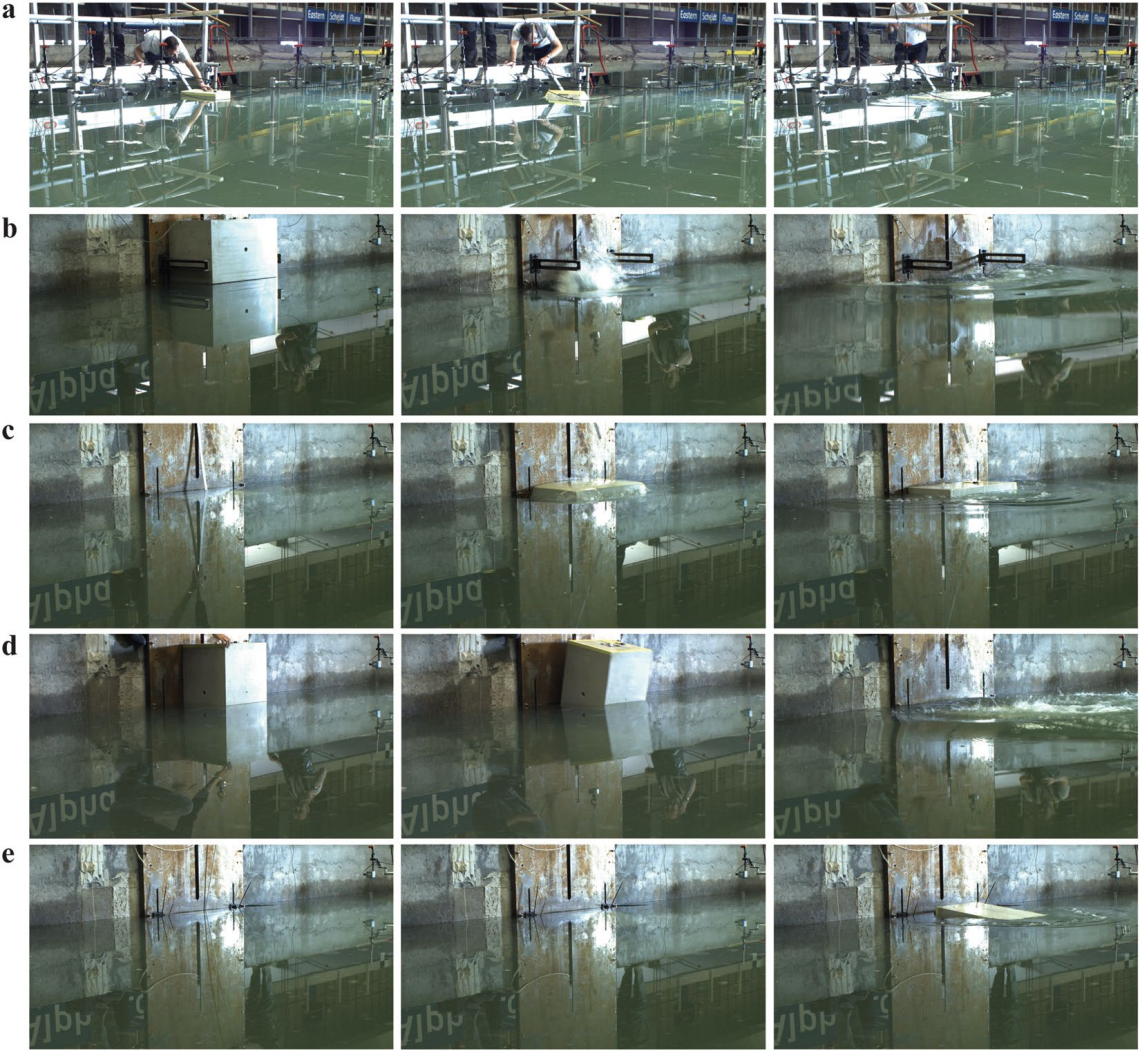
Image series of our large-scale experiments conducted in the basin at a water depth of 1.00 m. **(a)** Capsizing (mechanism A). **(b)** Gravity-dominated fall (B). **(c)** Buoyancy-dominated fall (C). **(d)** Gravity-dominated overturning (D). (**e**), Buoyancy-dominated overturning (E). The shown examples of mechanisms A and E were conducted with a 0.800 m × 0.500 m × 0.250 m block (type 2) and the two examples of mechanisms B, C and D with a 0.800 m × 0.500 m × 0.500 m block (type 1). Wave profiles and movies for the five mechanisms are shown in Fig. 4 and the Supplementary Information.

**Figure 3:**
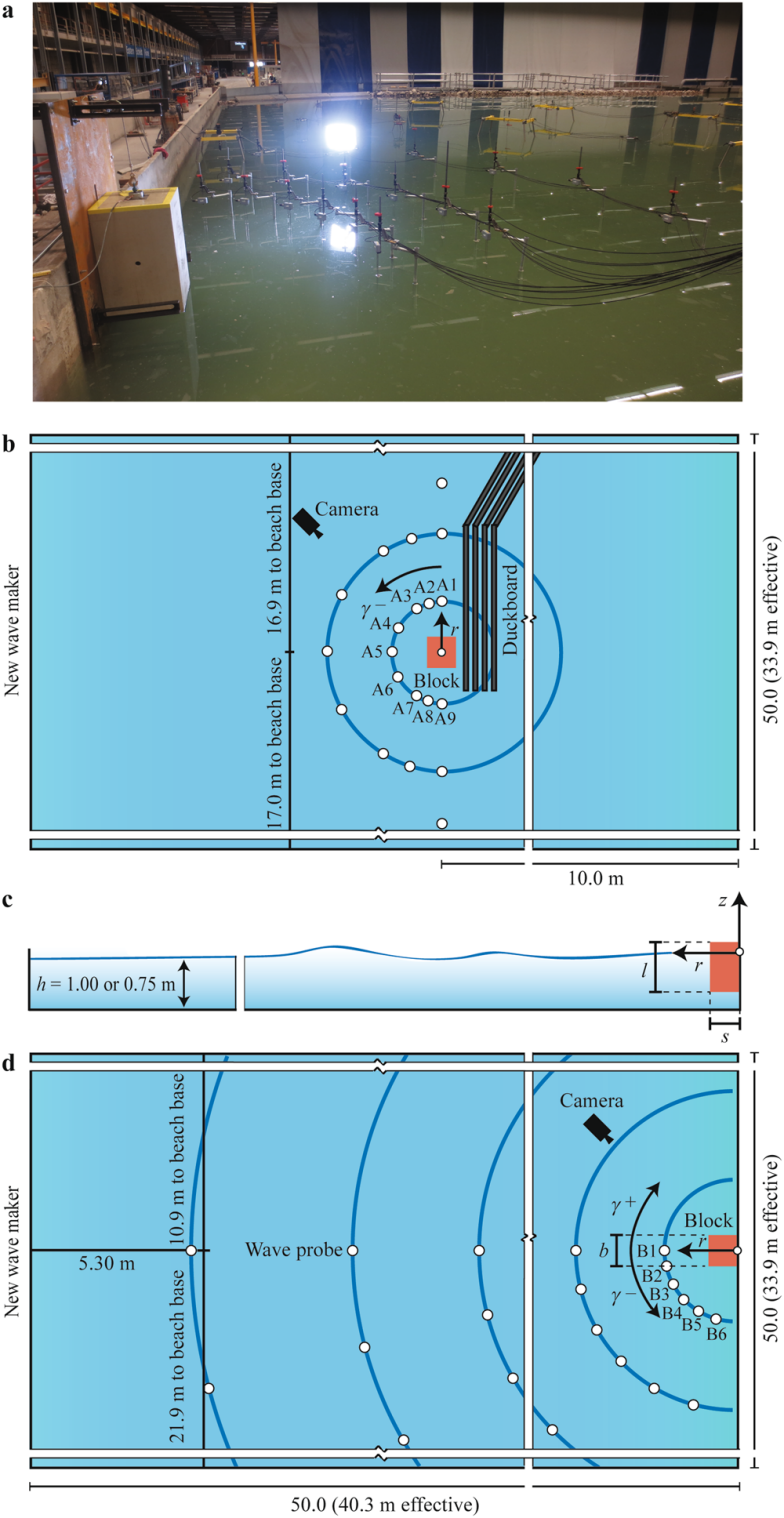
Experimental set-up. **(a)** Picture of iceberg block type 1 (0.800 m × 0.500 m × 0.500 m) in the gravity-dominated fall release position (mechanism B) at the wall of the 50 m × 50 m wave basin. **(b)** Plan view of capsizing case. The wave probes A1 to A9 at *r* = 2 *h* are located at *γ* = 0°, −15°, −30°, −60°, −90°, −120°, −150°, −165° and −180°. **(c)** Side view of a gravity-dominated fall experiment. **(d)** Plan view of a gravity-dominated fall experiment with wave probe locations. The wave probes B1 to B6 at *r* = 2 *h* are located at *γ* = 0°, −15°, −30°, −45°, −60° and −75°.

Unique large-scale experiments have been conducted in the 50 m × 50 m large wave basin at Deltares (Figs 2 and 3a). This large size basin allowed the tsunamis to propagate freely on an area of 40.3 m × 33.9 m between absorbing beaches and basin boundaries. A total of 66 experiments have been conducted at water depths of 1.00 m or 0.75 m, respectively. Experimental conditions are given in Table 1. The experiments involved 16 capsizing (mechanism A), 21 gravity-dominated fall (B), 9 buoyancy-dominated fall (C), 14 gravity-dominated overturning (D) and 6 buoyancy-dominated overturning mechanisms (E). Mechanism A was investigated offshore (Fig. 3b) and all other experiments were conducted at the vertical boundary of the basin (Fig. 3a,c,d).

**Table 1:** Experimental conditions.

Iceberg block parameter	Capsizing (mechanism A)			Fall (mechanisms B and C)						Overturning (mechanisms D and E)			
Block release location	Offshore	Offshore	Offshore	At shore	At shore	At shore	At shore	At shore	At shore	At shore	At shore	At shore	At shore
Block type	1	2	2	1	2	1	1	2	2	1	2	2	2
Block length *l* (m)	0.800	0.800	0.500	0.800	0.800	0.500	0.500	0.500	0.500	0.800	0.800	0.500	0.500
Block width *b* (m)	0.500	0.500	0.800	0.500	0.500	0.800	0.800	0.800	0.800	0.500	0.500	0.800	0.800
Block thickness *s* (m)	0.500	0.250	0.250	0.500	0.250	0.500	0.500	0.250	0.250	0.500	0.250	0.250	0.250
Block volume *V*_s_ (m^3^)	0.200	0.100	0.100	0.200	0.100	0.200	0.200	0.100	0.100	0.200	0.100	0.100	0.100
Block density *ρ*_*s*_ (kg/m^3^)	929	924	924	936/923	936/912	936/923	936/923	936/912	936/912	936/923	912	912	936/912
Mass *m*_*s*_ (kg)	185.8	92.4	92.3	187.1/184.6	93.6/91.2	187.1/184.6	187.1/184.6	93.6/91.2	93.6/91.2	187.1/184.6	91.2	91.2	93.6/91.2
Water depth *h* (m)	1.000	1.000	1.000	1.000	1.000	1.000	0.750	1.000	0.750	1.000	1.000	1.000	0.750
Release position above still water level (m)	Neutrally buoyant	Neutrally buoyant	Neutrally buoyant	0.00, −0.30, −0.60, −0.84	0.00, −0.30, −0.60, −0.83	0.30, 0.00, −0.30, −0.60, −0.70, −0.83	0.30, 0.00, −0.30, −0.60	0.30, 0.00, −0.30, −0.60, −0.83	0.30, 0.00, −0.30, −0.60	0.15, 0.00, −0.30, −0.60, −0.90	0.15, 0.00, −0.30, −0.60, −0.90	0.15, 0.00, −0.30, −0.60	0.15, 0.00, −0.30, −0.60
Number of runs	5^+^	6^+^	5^+^	6^+^	4	7^+^	4	5	4	5	5	4	6^+^

Overview of all investigated test parameters in the 66 experiments; The block densities changed slightly with the attachments to the blocks (rod, bearing, etc.); The number of runs indicated with ^+^ include test repetitions.

Icebergs were modelled with blocks consisting of polypropylene homopolymer (PPH) with a density similar to ice (≈920 kg/m^3^). The block sizes were 0.800 m × 0.500 m × 0.500 m (block type 1, Fig. 3a) and 0.800 m × 0.500 m × 0.250 m (block type 2) and weighed approximately 187 kg and 92 kg, respectively (Table 1).

### Calving mechanisms

The five iceberg calving mechanisms were controlled as follows; mechanism A (Figs 1c, 2a and 3b, Supplementary Movie S1): the blocks rotated relative to a wooden rod fed through the centres of the blocks. This rod allowed for rotation around the *y*-axis and translation in the *z*-direction only. The block capsized either naturally or under a small force of approximately 1 N. This force was increased in some experiments to accelerate the rotation and wave generation. Mechanism B (Figs 1c, 2b and 3a, Supplementary Movie S2): the blocks were held in position with an electromagnet via a winch system supported with a purpose-built steel frame which was fixed to the basin wall. Mechanism C (Figs 1c and 2c, Supplementary Movie S3): the blocks were pulled under water with a rope attached to the centre of the block bottom. In addition, the blocks were stabilised with a steel beam from above for some of the tests. Mechanisms D and E (Figs 1c and 2d,e, Supplementary Movies S4 and S5): the blocks were rotated around a fixed steel rod of 30 mm diameter. This rod was fed through two ball bearings attached to the block surface and allowed for rotation, but no translation. The rod was located either below (mechanism D) or above (mechanism E) the blocks. The blocks were stabilised with a steel beam from above (Fig. 2e) for some experiments of mechanism E.

### Mass kinematics and wave probes

The maximum block velocity *V*_*s*_ corresponding to the fastest moving section of the block was recorded with a 9 degree of freedom motion sensor. The sensor was attached to the block surfaces as shown in the Supplementary Movies S1 to S5 which were recorded with a 5 MP camera at 15 Hz. Wave profiles were recorded in different directions on one side of the block axis, given that the wave field is symmetric in relation to this axis, with resistance type wave gauges. The positions of the camera and wave probes are shown in Fig. 3b,d.

## Results

### Wave characteristics

**Figure 4:**
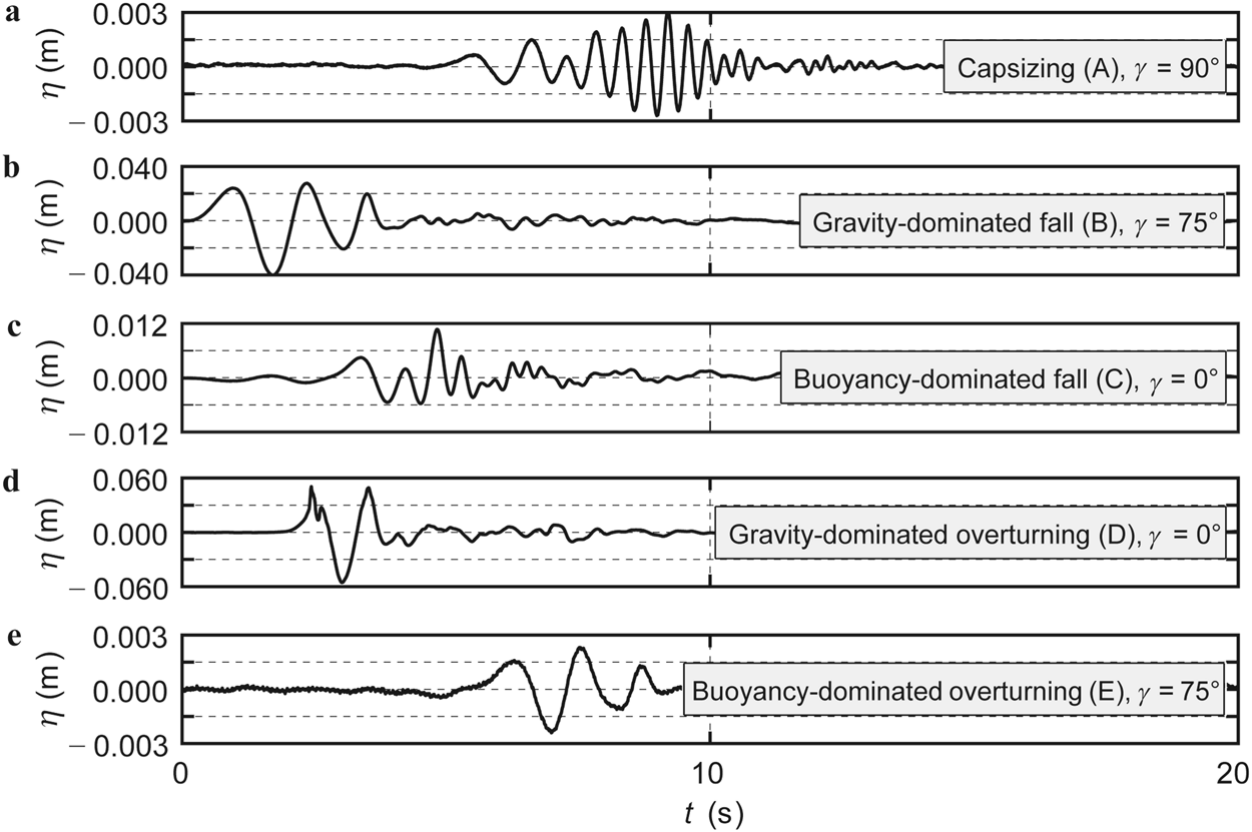
Iceberg-tsunami profiles of the five iceberg calving mechanisms A to E shown in Fig. 2. These tsunami profiles were recorded at (*r*/*h* = 2, *γ*) where the maximum wave height *H*_*M*_ was measured. **(a)** Capsizing mechanism A. **(b)** Gravity-dominated fall mechanism B. **(c)** Buoyancy-dominated fall mechanism C. **(d)** Gravity-dominated overturning mechanism D. **(e)** Buoyancy-dominated overturning mechanism E. The scale on the *y*-axes change by up to a factor of 20.

Wave characteristics, including the maximum wave height, are of primary relevance to understand iceberg-tsunamis and associated hazards. The free water surface *η* versus time *t* of the five experiments shown in Fig. 2 are presented in Fig. 4. These wave profiles were all measured at relative radial distance *r*/*h* = 2 from the origin with *r* specifying the radial coordinate and *h* the still water depth (Fig. 3b,d). Cylindrical coordinates are used to characterise the wave location as the waves propagate on a circle (Fig. 3b) or semi-circle (Fig. 3d) of radius *r* and wave propagation angle *γ*. The scales on the *y*-axes in Fig. 4 vary by up to a factor of 20. Significantly different wave heights in function of the mechanisms A to E are revealed; the gravity-dominated overturning mechanism D resulted in the largest tsunamis followed by the gravity-dominated fall mechanism B. The three remaining mechanisms resulted in up to a factor of 27 smaller waves.

The wave trains consist of several nonlinear waves for all mechanisms and show some similarities to subaerial landslide-tsunamis^25,26^. The largest wave is observed in the middle of the wave train for the slower moving mechanisms A, C and E. For the gravity-dominated mechanisms B and D the largest wave is observed earlier in the wave train, but not always at the first wave (Fig. 4).

### Released energy and maximum wave heights

An aim of this work is to experimentally quantify the maximum iceberg-tsunami heights as a function of the mechanisms A to E and the iceberg volume, geometry and kinematics (Table 1). A key parameter to quantify the tsunami features is the released energy *E* from the iceberg block to the surrounding water. It is convenient to link the tsunami features to this energy as an estimate of *E* can readily be derived from the geometry and position of the iceberg relative to the water surface, and no information about the speed of the iceberg movement is required. Released energy is transferred into the tsunami train, with losses in bobbing and rocking motions of the block and water system, viscous energy dissipation, friction losses in the experimental set-up (bearings, rod) and sometimes block impact on the basin floor in our laboratory experiments whilst in the field additional mechanisms such as the movement of the surrounding ice mélange^27^ or the mixing of the stratified water may consume additional energy^18,23^.

The released energy *E* of the blocks during capsizing was theoretically computed with an available method^23^. *E* is the difference between the work required to move the iceberg block in the initial (*W*_*i*_) and final (*W*_*f*_) positions to a common reference level above the water surface by considering gravity force and hydrostatic pressure force

1 $$E={W}_{i}-{W}_{f}=\frac{1}{2}b{\rho }_{i}\,gs{l}^{2}(1-s/l)(1-\frac{{\rho }_{i}}{{\rho }_{w}})$$

In equation (1) *b*, *l* and *s* are the block width, height and thickness (Fig. 3c,d), *g* is the gravitational acceleration and *ρ*_*i*_ and *ρ*_*w*_ is the ice and water density, respectively. We expanded this method for the capsizing mechanism A to all other mechanisms B to E. Graphical illustrations and the theoretical expressions for work and released energy are shown in the Supplementary Table S1.

**Figure 5:**
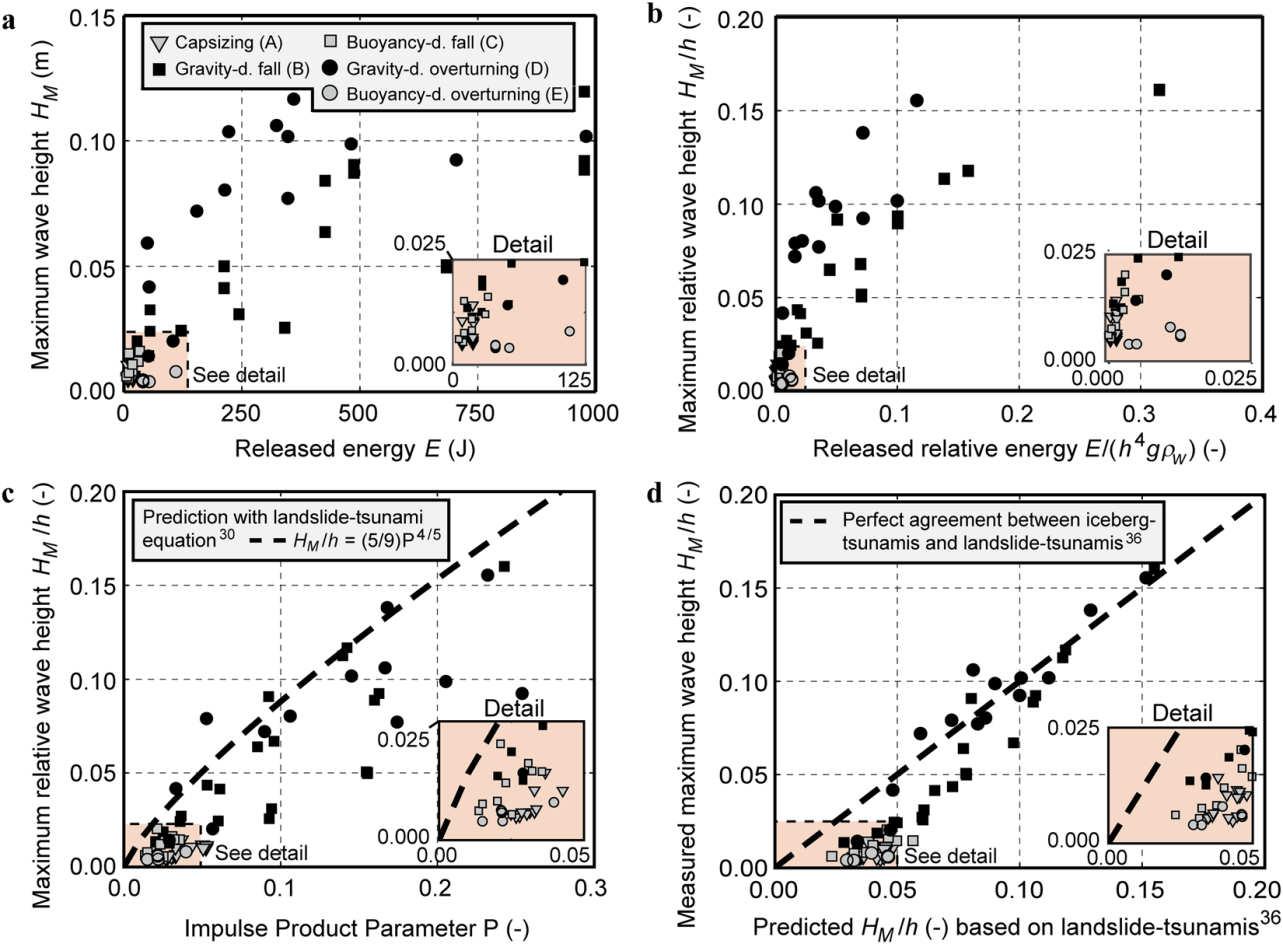
Measured maximum wave heights *H*_*M*_ for all 66 experiments. **(a)** *H*_*M*_ versus released block energy *E* showing that the gravity-dominated mechanisms B and D (black symbols) generate typically an order of magnitude larger tsunamis than the capsizing and buoyancy-dominated mechanisms A, C and E (grey symbols). **(b)** Relative maximum wave height *H*_*M*_/*h* versus dimensionless energy *E*/(*h*^4^*gρ*_*w*_) (for notation see a). **(c)** Measured *H*_*M*_/*h* versus Impulse Product Parameter P and (–) empirical equation for landslide-tsunamis^30^ resulting in an estimate of an upper envelope for all mechanisms (for notation see a). **(d)** Measured *H*_*M*_/*h* versus predicted *H*_*M*_/*h* based on empirical equation for landslide-tsunamis^36^ with (–) perfect agreement (for notation see a). The inserts show details of the data. The absolute and relative measurement errors are shown in Table 2. The shown data are included in the Supplementary Spreadsheet S1.

Released energy is then related in Fig. 5a to the measured maximum tsunami height *H*_*M*_, which was always observed at a wave probe location at *r*/*h* = 2 (Fig. 3b,d) for all five mechanisms. Figure 5a reveals that the gravity-dominated overturning mechanism D generates the largest waves followed by the gravity-dominated fall mechanism B, in agreement with theoretical predictions^20^. Mechanism D may generate larger tsunamis than B as the blocks move closer to the measurement location (Fig. 2). *H*_*M*_ of mechanism A are considerable smaller and reach 0.6 to 1.1% of the initial vertical dimension of the mass for naturally capsizing icebergs in good agreement with previous theoretical estimates of 1%^18,23^. The two remaining calving mechanisms C and E result in significantly smaller waves than mechanisms B and D. The released energy *E* supports this important finding; *E* is up to an order of magnitude larger for mechanisms B and D than for A, C and E. This notable result reveals that icebergs of a given volume and geometry released above the water surface are significantly more hazardous in terms of tsunami generation than neutrally buoyant icebergs or icebergs released underwater.

Data scatter significantly reduces in Fig. 5b where *H*_*M*_ and *E* are plotted in dimensionless form with the water depth *h*, gravitational acceleration *g* and water density *ρ*_*w*_ as reference quantities. The maximum relative wave height observed over all experiments is *H*_*M*_/*h* = 0.160 for the gravity-dominated mechanisms B and D and only *H*_*M*_/*h* = 0.020 for the remaining mechanisms. Limitations to avoid significant scale effects for the maximum landslide-tsunami amplitude have been formulated in terms of a limiting Reynolds number R = *g*^1/2^*h*^3/2^/*ν*_*w*_ ≥ 300,000 and Weber number W = *ρ*_*w*_*gh*^2^/*σ*_*w*_ ≥ 5,000^28^. For our iceberg-tsunami experiments 2,033,835 ≤ R ≤ 3,131,294 and 75,552 ≤ W ≤ 134,315, based on a kinematic viscosity *ν*_*w*_ = 10^−6^ m^2^/s and surface tension *σ*_*w*_ = 0.073 N/m at the water temperature of 19.3 °C in our experiments. The kinematic viscosity for a sea temperature of 0 °C approximately observed around Greenland is with *ν*_*w*_ = 1.8 × 10^−6^ m^2^/s lower than in the laboratory, improving the laboratory experiments to field similarity further as the lower temperature reduces R observed in nature by nearly a factor of two to R ≈ 2 − 39 × 10^9^ (at *h* = 125 to 800 m). Scale effects are therefore expected to be insignificant and the figures in Fig. 5b may directly be transferred to field conditions based on Froude scaling^29^.

For a water depth *h* ≈ 125 m observed in the fjord of the Eqip Sermia Glacier^15^, the investigated scenarios result in maximum wave heights of up to 20.0 m (mechanisms B and D) and 2.5 m for the three remaining mechanisms. The measured wave periods of the maximum wave heights are 0.36 to 2.09 s at laboratory scale (Fig. S1) corresponding to a period of up to 23.4 s in nature after Froude scaling^29^ (at scale 1:125). This results in a wavelength of 694 m by using the linear wave dispersion relation (Supplementary Methods). For a scale of 1:800 matching a water depth *h* ≈ 800 m for typical Greenlandic settings such as the Helheim Glacier^5^ and Jakobshavn Isbræ^27^, the maximum wave height of mechanisms B and D is unlikely to be reached due to the limited iceberg thickness above water. However, mechanisms A, C and E would be predicted to result in a maximum wave height of up to 16.0 m. The corresponding maximum period is 59.1 s (4431 m wavelength), and is found to be in agreement with measured wave periods of 30–60 s in the field^14^. Most iceberg-tsunami periods are thus much larger than for typical gravity ocean waves (10 s), and show similarities to the lower spectrum of landslide-tsunamis^25,26,30,31^.

### Tsunami train energy

The energy *E*_*w*_ of the wave train passing the circle (Fig. 3b) or semi-circle (Fig. 3d), respectively, located at *r*/*h* = 2 was calculated with the method given in the Supplementary Information. The energy *E*_*w*_ accounts for 0.6 to 56.9% of the released energy *E* over all mechanisms. Bobbing and rocking motions of the block and water system, viscous energy dissipation, friction losses in the experimental set-up and block impact on the basin floor tend to consume most of the released energy *E*. The most efficient wave generator is the gravity-dominated fall mechanism B (4.7 to 56.9% of *E* becomes contained in the wave train) followed by the gravity-dominated overturning mechanism D (2.4 to 41.8%), buoyancy-dominated fall mechanism C (5.1 to 18.6%) and natural capsizing mechanism A (2.8 to 5.0%). The buoyancy-dominated overturning mechanism E is the most inefficient wave generator (0.6 to 1.0%). The values for the naturally capsizing cases (2.8 to 5.0%) are significantly larger than 1% found for iceberg-tsunami trains in confined small flume experiments^18^. The efficiencies for the gravity-dominated fall mechanisms (4.7 to 56.9%) are similar to solid subaerial landslide-tsunamis generated in a confined flume where 18 and 47%^32^ and 6 to 40%^33^ of the kinetic slide energy was converted to the primary wave, but larger than for granular slides impacting into a wave basin where only 1 to 15% of the kinetic slide energy was converted into the wave train^31^. Icebergs interact with the surrounding water more efficiently than granular slides which dissipate energy due to internal and basal friction as well as the impact on the flume or basin floor.

### Comparison with landslide-tsunamis

Our present knowledge of iceberg-tsunamis relies mainly on field observations^7,14,15,17,19,21^, theoretical work^20,22,23^ and small flume experiments^18^. In order to potentially transfer knowledge from the significantly further advanced landslide-tsunami research field, we link our results to subaerial landslide-tsunamis^25,26,28,30–38^. In addition, the measured maximum wave heights are compared with empirical landslide-tsunami height prediction equations^30,36^ in Fig. 5c,d. Figure 5c shows *H*_*M*_/*h* versus the Impulse Product Parameter P, developed for landslide-tsunamis^30^, given as

2 $${\rm{P}}={\rm{F}}{S}^{1/2}{M}^{1/4}{\{\cos [(\frac{6}{7})\alpha ]\}}^{1/2}$$

F = *V*_*s*_/(*gh*)^1/2^ in equation (2) is the slide Froude number with the slide impact velocity *V*_*s*_, the gravitational acceleration *g* and the water depth *h*, *S* = *s*/*h* is the relative slide thickness with the slide thickness *s*, *M* = *m*_*s*_/(*ρ*_*w*_*bh*^2^) is the relative slide mass with the slide mass *m*_*s*_, the water density *ρ*_*w*_ and the slide width *b* and *α* is the hill slope angle. The slide impact velocity is represented by the maximum block velocity 0.27 ≤ *V*_*s*_ ≤ 4.17 m/s in our study and all slide parameters are replaced by the corresponding iceberg block parameters shown in Table 1 resulting in 0.09 ≤ F ≤ 1.33, 0.25 ≤ *S* ≤ 0.67, 0.11 ≤ *M* ≤ 0.42, *α* = 90° and 0.01 ≤ P ≤ 0.32. Measurement errors for these parameters are shown in Table 2. The parameter limitations in the original study^30^ can be found in the Supplementary Methods; the experiments included slide densities lighter than water and vertical shores (*α* = 90°). However, they were conducted with granular slides impacting into a flume^30^ with a similar geometry as the first section of the Helheim glacier fjord^21^ and small-scale iceberg-tsunami experiments^18^. Granular rather than solid slides and a flume rather than a basin geometry are potential reasons for deviations between the measurements and the predictions in Fig. 5c^25,26,34,37^.

**Table 2:** Absolute and relative measurement errors^39^.

Iceberg block parameter	Absolute errors (top) and relative errors (bottom)
Water depth *h*	Δ*h* = ±0.003 m
Block thickness *s*	Δ*s* = ±0.001 m
Block width *b*	Δ*b* = ±0.001 m
Block length *l*	Δ*l* = ±0.001 m
Mass *m*_*s*_	Δ*m*_*s*_ = ±0.050 kg
Block velocity *V*_*s*_	Δ*V*_*s*_ = ±0.05 m/s^A^; Δ*V*_*s*_ = ± 0.08 m/s^B^; Δ*V*_*s*_ = ± 0.03 m/s^C^; Δ*V*_*s*_ = ± 0.05 m/s^D^; Δ*V*_*s*_ = ± 0.03 m/s^E^
Slope angle *α*	Δ*α* = ±1.0°
Maximum wave period *T*_*M*_	Δ*T*_*M*_ = ±0.03s
Maximum wave height *H*_*M*_ and amplitude *a*_*M*_	Δ*Η*_*Μ*_ = Δ*a*_*Μ*_ = ± 0.002 m (for tsunamis affected by air and splash); Δ*Η*_*Μ*_ = Δ*a*_*Μ*_ = ±0.0002 m (for pure water tsunamis)
Froude number F = *V*_*s*_/(*gh*)^1/2^	$\frac{{\rm{\Delta }}{\rm{F}}}{|{\rm{F}}|}=\sqrt{{(\frac{{\rm{\Delta }}{V}_{s}}{{V}_{s}})|}^{2}+{(\frac{{\rm{\Delta }}g}{2g})}^{2}+{(\frac{{\rm{\Delta }}h}{2h})}^{2}}$ = ±0.097^A^; ±0.230^B^; ±0.110^C^; ±0.085^D^; ±0.094^E^
Relative block thickness *S* = *s*/*h*	$\frac{{\rm{\Delta }}S}{|S|}=\sqrt{{(\frac{{\rm{\Delta }}S}{S})}^{2}+{(\frac{{\rm{\Delta }}h}{h})}^{2}}$ = ±0.006
Relative mass *M = m*_*s*_/(*ρ*_*w*_*bh*^2^)	$\frac{{\rm{\Delta }}M}{|M|}=\sqrt{{(\frac{{\rm{\Delta }}{m}_{s}}{{m}_{s}})}^{2}+{(\frac{{\rm{\Delta }}{\rho }_{w}}{{\rho }_{w}})}^{2}+{(\frac{{\rm{\Delta }}b}{b})}^{2}+{(\frac{2{\rm{\Delta }}h}{h})}^{2}}$ = ±0.008
Impulse Product Parameter P (equation (2))	$\frac{{\rm{\Delta }}{\rm{P}}}{|{\rm{P}}|}=\sqrt{{(\frac{{\rm{\Delta }}{V}_{s}}{{V}_{s}})}^{2}+{(\frac{{\rm{\Delta }}s}{2s})}^{2}+{(\frac{{\rm{\Delta }}{m}_{s}}{4{m}_{s}})}^{2}+{(\frac{3\tan [(6/7)\alpha ]{\rm{\Delta }}\alpha }{7})}^{2}+{(\frac{{\rm{\Delta }}g}{2g})}^{2}+{(\frac{3{\rm{\Delta }}h}{2h})}^{2}+{(\frac{{\rm{\Delta }}{\rho }_{w}}{4{\rho }_{w}})}^{2}+{(\frac{{\rm{\Delta }}b}{4b})}^{2}}$= ±0.103^A^; ±0.232^B^; ±0.115^C^; ±0.091^D^; ±0.100^E^
Maximum relative wave height *H*_*M*_/*h*	${\rm{\Delta }}(\frac{{H}_{M}}{h})/|\frac{{H}_{M}}{h}|=\sqrt{{(\frac{{\rm{\Delta }}{H}_{M}}{{H}_{M}})}^{2}+{(\frac{{\rm{\Delta }}h}{h})}^{2}}$ = ±0.051 (for all tsunamis)

The absolute errors are shown on the top and their propagation into the dimensionless parameters on the bottom resulting in the specified relative errors. The superscripts A, B, C, D and E refer to the five iceberg calving mechanisms. The largest uncertainty is associated with the block velocity. The measurement errors for the water density and gravitational acceleration are set as Δ*ρ*_*w*_ = Δ*g* = 0. Most of the uncertainties for F, *S*, *M*, P and *H*_*M*_/*h* for the individual experiments are significantly smaller than the specified values for the most uncertain experiments.

Figure 5d shows the measured versus the predicted relative maximum wave heights *H*_*M*_/*h* based on landslide-tsunami experiments^36^ conducted in a basin with mesh-packed granular material including tests with *α* = 90° (Supplementary Methods). A main difference of the landslide-tsunami experiments^36^ compared to our study is that a larger bulk slide density of 1338 kg/m^3^ was used, which may be the reason for the systematic overprediction of small wave heights in Fig. 5d. Overall, the gravity-dominated mechanisms B and D are clearly better predicted by landslide-tsunami models^30,36^ than the capsizing A and buoyancy-dominated mechanisms C and E. This was expected given that the physics of mechanisms A, C and E are very different from B, D and thus, subaerial landslide-tsunamis. Both methods^30,36^ are valuable in the sense that they establish estimates of an upper envelope for the maximum iceberg-tsunami heights.

## Discussion

Quantification of the maximum wave height as a function of the iceberg calving mechanism is important to protect coastal infrastructure and vessels navigating in proximity of glacier calving fronts. Our results reveal that iceberg-tsunamis generated by the gravity-dominated mechanisms B and D can be more than an order of magnitude larger than of capsizing or buoyancy-dominated processes for a given iceberg volume and geometry. However, not considered in this comparison is the fact that icebergs may move in proximity of a critical location, e.g. in front of a harbour, such that the significantly smaller iceberg-tsunamis originating from capsizing still resulted in large destruction in the recent past^24^. Further, deviations of the idealised conditions investigated herein including the iceberg geometry, the water body geometry and the coastal geometry and bathymetry will also significantly affect the iceberg-tsunamis^23,25,34,37,38^.

The 50 m large iceberg-tsunami observed in 2014 at Eqip Sermia^15^ (mechanism B) was successfully replicated with a landslide-tsunami hazard assessment method^38^. This motivated us to compare the measured maximum iceberg-tsunami heights with empirical equations based on landslide-tsunamis. Whilst the empirical equations of landslide-tsunamis^30,36^ are able to provide estimates of an upper envelope for the maximum iceberg-tsunami heights, they fail to predict the behaviour of the capsizing A and buoyancy-dominated mechanisms C and E (Fig. 5c,d). Additional, empirical landslide-tsunami equations^25,26,31^ were found to be less capable in predicting iceberg-tsunamis than the two selected equations^30,36^, probably because their experimental conditions are significantly different from our experiments (Table 1). Whilst knowledge from the significantly further advanced landslide-tsunami research field may help to give initial estimates for iceberg-tsunamis, particularly for mechanism B^15^, transferred knowledge from landslide-tsunamis cannot replace the requirement to further study iceberg-tsunamis.

## Conclusions

Unique large-scale experiments have been conducted in a 50 m × 50 m wave basin to investigate iceberg-tsunamis with up to 187 kg heavy blocks under variation of the iceberg volume, geometry and kinematics. The blocks interacted with the surrounding water through five iceberg calving mechanisms A: capsizing, B: gravity-dominated fall, C: buoyancy-dominated fall, D: gravity-dominated overturning and E: buoyancy-dominated overturning.

The tsunami heights generated by mechanisms B and D (gravity-dominated) were roughly an order of magnitude larger than from mechanisms A, C and E. A theoretical model for the capsizing case was applied to the remaining mechanisms to compute the released iceberg energy, supporting that gravity-dominated iceberg calving generate the largest waves. However, only between 0.6 to 56.9% of the released energy is transferred into the wave train with the rest lost in other processes. Results were upscaled to Greenlandic outlet glaciers and the wave periods agree well with field observations. The iceberg-tsunamis were also predicted with empirical equations for landslide-tsunamis resulting in a good match for some gravity dominated cases and estimates of an upper envelope of the maximum iceberg-tsunami heights over all mechanisms. However, these equations fail to capture the physics of most iceberg-tsunami mechanisms such that the new research field of iceberg-tsunamis requires more attention.

## Electronic supplementary material

Supplementary Methods

Movie S1

Movie S2

Movie S3

Movie S4

Movie S5

Spreadsheet S1

## Data Availability

The raw data of this study is available from http://hydralab.eu/ and the processed data is included in the Supplementary Spreadsheet S1.
