# Supplementary material for: Large-scale experiments into the tsunamigenic potential of different iceberg calving mechanisms
Source: Sci Rep. 2019 Jan 29;9:861. doi: 10.1038/s41598-018-36634-3 (PMC6351686; doi:10.1038/s41598-018-36634-3)
Supplement: Supplementary file 1 — Supplementary Methods [file 41598_2018_36634_MOESM1_ESM.pdf]

Large-scale experiments into the tsunamigenic potential of different iceberg calving mechanisms

Valentin Heller, Fan Chen, Markus Brühl, Roman Gabl, Xuexue Chen, Guido Wolters and Helge Fuchs

Supplementary Methods

Tsunami train energy. The energy E_w of the wave train passing the circle (Fig. 3b) or semi-circle (Fig. 3d), respectively, located at $r/h = 2$ was calculated as

$$E_w = \sum_{i,j=1}^{n,m} E_{wij} \qquad \text{(S1)}$$

where n specifies the number of wave probes and m the number of waves in the wave train. We estimated the wave energy E_{wij} for each sector i represented by one wave probe and the jth wave in the wave train with the linear wave expression[S1]

$$E_{wij} = \frac{1}{8}\rho_w g H_{ij}^2 L_{ij} \Delta_i \pi r \qquad \text{(S2)}$$

The wave height H_{ij} was measured and the wavelength L_{ij} was calculated with the linear dispersion relation

$$L_{ij} = \frac{g}{2\pi} T_{ij}^2 \tanh\left(2\pi \frac{h}{L_{ij}}\right) \qquad \text{(S3)}$$

based on the measured wave period T_{ij}. The term πr in equation (S2) considers that the investigated iceberg-tsunamis propagated on circles (mechanism A, Fig. 3b) or semi-circles (mechanism B to E, Fig. 3d), respectively, from the source. Every wave probe (A1 to A9 ($n = 9$) for mechanism A shown in Fig. 3b; B1 to B6 ($n = 6$) for mechanisms B to E shown in Fig. 3d) contributed to the overall energy E_w according to the measured wave parameters H_{ij} and T_{ij} at each probe location and the radial angle $\Delta_i\pi = \pi/12$ (for probes A1, A9 and B1), $\Delta_i\pi = \pi/6$ (A2, A8, B2, B3, B4, B5), $\Delta_i\pi = 3\pi/12$ (A3, A7, B6) and $\Delta_i\pi = \pi/3$ (A4, A5, A6).

Empirical equation for landslide-tsunamis[30]. 211 subaerial landslide-tsunami experiments with granular slides impacting into a 11 m long, 0.5 m wide and 1.0 m deep flume have been conducted[30]. The wave features were measured in the range $2.7 \leq x/h \leq 59$ with seven capacitance type wave gauges. The maximum relative wave height H_M/h computes as

$$\frac{H_M}{h} = \left(\frac{5}{9}\right) \mathrm{P}^{4/5} \tag{S4}$$

with the Impulse Product Parameter P given in equation (2). The parameter limitations for the water depth are $0.150 \leq h \leq 0.675$ m, for the slide thickness $0.050 \leq s \leq 0.249$ m, for the slide impact velocity $2.06 \leq V_s \leq 8.77$ m/s, for the slide mass $10.09 \leq m_s \leq 113.30$ kg, for the hill slope angle $30 \leq \alpha \leq 90°$, for the Froude number $0.86 \leq \mathrm{F} \leq 6.83$, for the relative slide thickness $0.09 \leq S \leq 1.64$, for the relative slide mass $0.11 \leq M \leq 10.02$ and $0.17 \leq \mathrm{P} \leq 8.13$. The experiments included bulk slide densities in the range of 590 to 1720 kg/m^3.

Empirical equation for landslide-tsunamis[36]. 74 subaerial landslide-tsunami experiments with mesh-packed granular slides in an 8.0 m wide, 4.5 m long and 0.75 m deep basin have been conducted[36]. The wave features were measured in the range $1.1 \leq r/h \leq 16.3$ with a videometric measurement system allowing for a quasi-continuous representation of the free water surface. The relative initial first wave height H_M/h computes as

$$\frac{H_M}{h} = 0.2\mathrm{P}^{0.5} B^{0.75} \left\{\cos\left[\left(\frac{6}{7}\right)\alpha\right]\right\}^{0.25} + 0.35\left(\mathrm{P}B\left\{\cos\left[\left(\frac{6}{7}\right)\alpha\right]\right\}\right)^{0.50} \tag{S5}$$

The first term on the right in equation (S5) corresponds to the wave crest and the second term to the wave trough. $B = b/h$ is the relative slide width and the notation of the remaining parameters is consistent with the notation in the previous section. The parameter limitations are $0.20 \leq h \leq 0.40$ m, $0.06 \leq s \leq 0.12$ m, $0.72 \leq V_s \leq 4.76$ m/s, $10 \leq m_s \leq 40$ kg, $30 \leq \alpha \leq 90°$, $0.40 \leq \mathrm{F} \leq 3.40$, $0.15 \leq S \leq 0.60$, $0.25 \leq M \leq 1.00$ and $0.13 \leq \mathrm{P} \leq 2.08$. All experiments were conducted with a bulk slide density of 1338 kg/m^3.

Reference

S1. Dean, R.G. & Dalrymple, R.A. *Water wave mechanics for engineers and scientists*. (World Scientific Publishing Co., 1991).

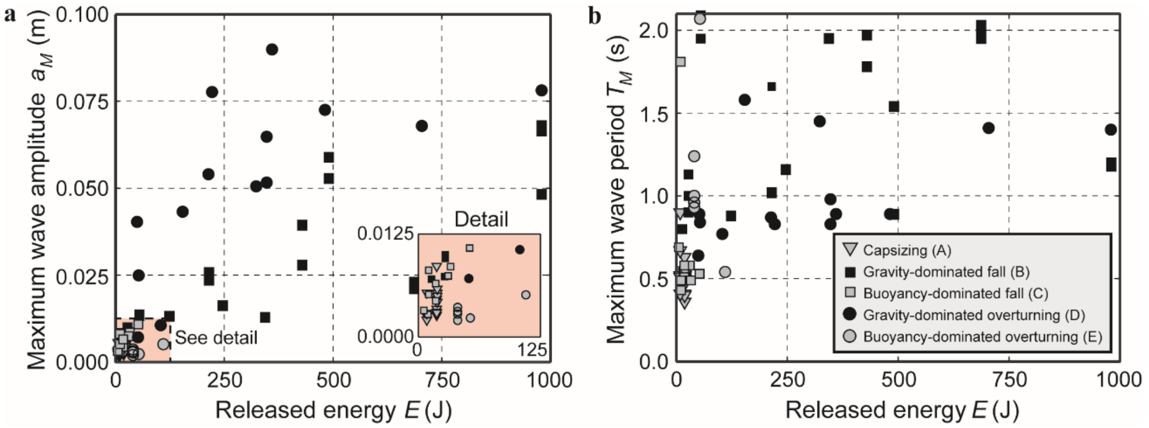

Supplementary Figure S1 | Maximum wave parameters. a, maximum wave amplitude a_M versus the released block energy E. The gravity-dominated overturning mechanism D generates the largest wave amplitudes. The insert shows a detail of the data (for notation see b). **b,** wave period T_M of H_M versus the released block energy E. No clear trend for the wave periods as a function of the iceberg-calving mechanism is revealed. The absolute and relative measurement errors are included in Table 2. The shown data is included in the Supplementary Spreadsheet S1.

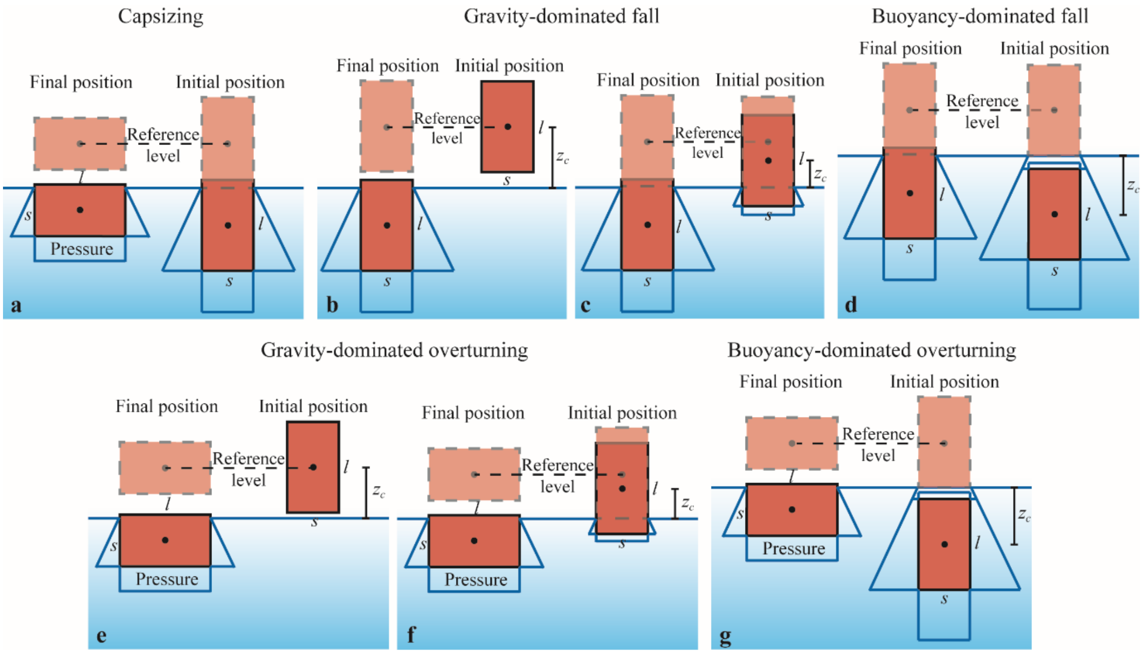

Supplementary Figure S2 | Illustration of five iceberg-calving mechanisms as base for the theoretically released energy calculation in Table S1. a, capsizing. **b,** gravity-dominated fall (block initially out of water). **c,** gravity-dominated fall (block initially in water). **d,** buoyancy-dominated fall. **e,** gravity-dominated overturning (block initially out of water). **f,** gravity-dominated overturning (block initially in water). **g,** buoyancy-dominated overturning.

Supplementary Table S1 | Theoretical released energy for the five iceberg-calving mechanisms based on Fig. S2. We applied an existing method[23] for capsizing to all calving mechanisms A to E herein. The released energy $E = W_i - W_f$ is the difference between the work required to lift the block in the initial (W_i) and the final positions (W_f) to a common reference level above the water surface. The block dimensions are l (height) × s (thickness) × b (width) (Fig. 3). The parameter ρ_i is the ice density, ρ_w the water density, g gravitational acceleration and z is the vertical coordinate pointing upwards with the origin located on the still water surface with z_c as the vertical position of the block centre in the initial position.

Iceberg calving mechanism	Work W_i in the initial position of the block, work W_f in the final position of the block and released energy E
A: capsizing (Fig. S2a)	$W_i = -b\rho_i gsl\int_{-l\left(\frac{\rho_i}{\rho_w}-\frac{1}{2}\right)}^{\frac{l}{2}}\partial z - b\rho_w gs\int_{-l\frac{\rho_i}{\rho_w}}^{0} z\partial z = -\frac{1}{2}b\rho_i gsl^2\frac{\rho_i}{\rho_w}$<br>$W_f = -b\rho_i gsl\int_{-s\left(\frac{\rho_i}{\rho_w}-\frac{1}{2}\right)}^{\frac{l}{2}}\partial z - b\rho_w gl\int_{-s\frac{\rho_i}{\rho_w}}^{0} z\partial z = -\frac{1}{2}b\rho_i gsl\left(l + s\frac{\rho_i}{\rho_w} - s\right)$<br>$E = W_i - W_f = \frac{1}{2}b\rho_i gsl^2(1 - s/l)\left(1 - \frac{\rho_i}{\rho_w}\right)$
B: gravity-dominated fall (Fig. S2b)	$E = -W_f = b\rho_i gsl\int_{-l\left(\frac{\rho_i}{\rho_w}-\frac{1}{2}\right)}^{z_c}\partial z + b\rho_w gs\int_{-l\frac{\rho_i}{\rho_w}}^{0} z\partial z = b\,\rho_i gsl\left[z_c + \frac{l}{2}\frac{\rho_i}{\rho_w} - \frac{l}{2}\right]$
B: gravity-dominated fall (Fig. S2c)	$W_i = -b\rho_i gsl\int_{z_c}^{\frac{l}{2}}\partial z - b\rho_w gs\int_{z_c-\frac{l}{2}}^{0} z\partial z = -b\rho_i gsl\left[\frac{l}{2} - z_c\right] + \frac{1}{2}b\rho_w gs\left[z_c^2 - z_c l + \frac{l^2}{4}\right]$<br>$W_f = -b\rho_i gsl\int_{-l\left(\frac{\rho_i}{\rho_w}-\frac{1}{2}\right)}^{\frac{l}{2}}\partial z - b\rho_w gs\int_{-l\frac{\rho_i}{\rho_w}}^{0} z\partial z = -\frac{1}{2}b\rho_i gsl^2\frac{\rho_i}{\rho_w}$<br>$E = W_i - W_f = -b\rho_i gsl\left[\frac{l}{2} - z_c - \frac{l}{2}\frac{\rho_i}{\rho_w}\right] + \frac{1}{2}b\rho_w gs\left[z_c^2 - z_c l + \frac{l^2}{4}\right]$
C: buoyancy-dominated fall (Fig. S2d)	$W_i = -b\rho_i gsl\int_{z_c}^{\frac{l}{2}}\partial z - b\rho_w gs\int_{z_c-\frac{l}{2}}^{0} z\partial z + b\rho_w gs\int_{z_c+\frac{l}{2}}^{0} z\partial z = -b\rho_i gsl\left[\frac{l}{2} - z_c\right] - b\rho_w gsz_c l$<br>$W_f = -b\rho_i gsl\int_{-l\left(\frac{\rho_i}{\rho_w}-\frac{1}{2}\right)}^{\frac{l}{2}}\partial z - b\rho_w gs\int_{-l\frac{\rho_i}{\rho_w}}^{0} z\partial z = -\frac{1}{2}b\rho_i gsl^2\frac{\rho_i}{\rho_w}$<br>$E = W_i - W_f = -b\rho_i gsl\left[\frac{l}{2} - z_c - \frac{l}{2}\frac{\rho_i}{\rho_w}\right] - b\rho_w gsz_c l$

D: gravity-dominated over-turning (Fig. S2e)	$E = -W_f = b\rho_i gls \int_{-s\left(\frac{\rho_i}{\rho_w}-\frac{1}{2}\right)}^{z_c} \partial z + b\rho_w gl \int_{-s\frac{\rho_i}{\rho_w}}^{0} z\partial z = b\rho_i gls \left[z_c + \frac{s}{2}\frac{\rho_i}{\rho_w} - \frac{s}{2}\right]$
D: gravity-dominated over-turning (Fig. S2f)	$W_i = -b\rho_i gsl \int_{z_c}^{\frac{l}{2}} \partial z - b\rho_w gs \int_{z_c-\frac{l}{2}}^{0} z\partial z = -b\rho_i gsl \left[\frac{l}{2} - z_c\right] + \frac{1}{2} b\rho_w gs \left[z_c^2 - z_c l + \frac{l^2}{4}\right]$<br>$W_f = -b\rho_i gsl \int_{-s\left(\frac{\rho_i}{\rho_w}-\frac{1}{2}\right)}^{\frac{l}{2}} \partial z - b\rho_w gl \int_{-s\frac{\rho_i}{\rho_w}}^{0} z\partial z = -b\rho_i gsl \left[\frac{l}{2} + \frac{s}{2}\frac{\rho_i}{\rho_w} - \frac{s}{2}\right]$<br>$E = W_i - W_f = -b\rho_i gsl \left[-z_c + \frac{s}{2}\frac{\rho_i}{\rho_w} - \frac{s}{2}\right] + \frac{1}{2} b\rho_w gs \left[z_c^2 - z_c l + \frac{l^2}{4}\right]$
E: buoyancy-dominated over-turning (Fig. S2g)	$W_i = -b\rho_i gsl \int_{z_c}^{\frac{l}{2}} \partial z - b\rho_w gs \int_{z_c-\frac{l}{2}}^{0} z\partial z + b\rho_w gs \int_{z_c+\frac{l}{2}}^{0} z\partial z = -b\rho_i gsl \left[\frac{l}{2} - z_c\right] - b\rho_w gsz_c l$<br>$W_f = -b\rho_i gsl \int_{-s\left(\frac{\rho_i}{\rho_w}-\frac{1}{2}\right)}^{\frac{l}{2}} \partial z - b\rho_w gl \int_{-s\frac{\rho_i}{\rho_w}}^{0} z\partial z = -b\rho_i gsl \left[\frac{l}{2} + \frac{s}{2}\frac{\rho_i}{\rho_w} - \frac{s}{2}\right]$<br>$E = W_i - W_f = -b\rho_i gsl \left[-z_c + \frac{s}{2}\frac{\rho_i}{\rho_w} - \frac{s}{2}\right] - b\rho_w gsz_c l$
